# Natural-Based Biomaterials for Peripheral Nerve Injury Repair

**DOI:** 10.3389/fbioe.2020.554257

**Published:** 2020-10-16

**Authors:** Benedetta E. Fornasari, Giacomo Carta, Giovanna Gambarotta, Stefania Raimondo

**Affiliations:** ^1^Department of Clinical and Biological Sciences, University of Turin, Turin, Italy; ^2^Neuroscience Institute Cavalieri Ottolenghi, University of Turin, Turin, Italy

**Keywords:** biopolymer, tissue engineering, peripheral nerve repair, nerve guidance conduit, natural biomaterial

## Abstract

Peripheral nerve injury treatment is a relevant problem because of nerve lesion high incidence and because of unsatisfactory regeneration after severe injuries, thus resulting in a reduced patient’s life quality. To repair severe nerve injuries characterized by substance loss and to improve the regeneration outcome at both motor and sensory level, different strategies have been investigated. Although autograft remains the gold standard technique, a growing number of research articles concerning nerve conduit use has been reported in the last years. Nerve conduits aim to overcome autograft disadvantages, but they must satisfy some requirements to be suitable for nerve repair. A universal ideal conduit does not exist, since conduit properties have to be evaluated case by case; nevertheless, because of their high biocompatibility and biodegradability, natural-based biomaterials have great potentiality to be used to produce nerve guides. Although they share many characteristics with synthetic biomaterials, natural-based biomaterials should also be preferable because of their extraction sources; indeed, these biomaterials are obtained from different renewable sources or food waste, thus reducing environmental impact and enhancing sustainability in comparison to synthetic ones. This review reports the strengths and weaknesses of natural-based biomaterials used for manufacturing peripheral nerve conduits, analyzing the interactions between natural-based biomaterials and biological environment. Particular attention was paid to the description of the preclinical outcome of nerve regeneration in injury repaired with the different natural-based conduits.

## Introduction

Peripheral nerve repair outcome after an injury is often poor, indeed it has been estimated that only 3% of patients recover sensibility while the motor function is recovered by less than 25% of patients (Houshyar et al., 2019).

Peripheral nerve repair and the consequent recovery of sensory and/or motor function is a great challenge for both researchers in biomedical sciences and bioengineering, but also for clinicians. Peripheral nerve injuries can be repaired through surgical techniques, however, when a nerve injury with loss of substance (>5 mm) occurs, its repair involves the use of grafts of different origin, among which nerve guidance conduits (NGC) (Meek and Coert, 2008) which reduce myofibroblast infiltration and scar tissue formation, and physically support regenerating nerves (Lundborg, 2000).

In the last 10 years, a progressive increase in the number of publications concerning the use of new artificial conduits to promote peripheral nerve regeneration has been reported, demonstrating a greater interest in this field by researchers and clinicians. Indeed, there is an increase in the number of publications dealing with conduits for nerve regeneration over the years: a PubMed search, using the string “conduit OR tube OR (“Tissue Scaffolds”[Mesh]) AND “Nerve Regeneration”[Mesh],” delivered 409 results for the period 2000–2009 and 989 results for the period 2010–2019.

The use of conduits to bridge a nerve gap has shown promising results, but clinical applications are limited to the reconstruction of short nerve lesions (Wang and Sakiyama-Elbert, 2019). Indeed, different artificial nerve conduits are commercially available and approved by the FDA (US Food and Drug Administration) (Kehoe et al., 2012), but no implant is approved and available for 3.0cm or longer nerve defect, the injury length usually considered critical. The most frequently used FDA-approved natural-based biomaterials are collagen (NeuraGen^®^, Neuroflex^®^, NeuroMatrix^®^, RevolNerv^®^) and chitosan (Reaxon^®^), as recently accurately reviewed by Kornfeld et al. (2018). All marketed and FDA approved nerve conduits demonstrate satisfying recovery, but with some side effects or regeneration failure (Kornfeld et al., 2018). Therefore, further researches are necessary to develop new conduits that lead to better outcomes.

Clinical trials in the field of peripheral nerve regeneration must be preceded by long-lasting preclinical research, which usually starts with the manufacturing of a biomaterial with precise characteristics (Archibald et al., 1991; Hashimoto et al., 2002; Ahmed et al., 2003; Jansen et al., 2004; Sundback et al., 2004; Yang et al., 2007a; Xie et al., 2008; He et al., 2009). When finalized, this biomaterial is primarily tested on *in vitro* models to assess its biocompatibility, cytotoxicity, genotoxicity, the absence of toxic degradation products, the interaction between cells and the biomaterial, its degradation rate, cell proliferation upon the biomaterial and so on. *In vitro* assays are more easily reproducible and standardized, they also allow to reduce the number of animals used for *in vivo* experiments, following the “3R” philosophy (Reduce, Replace, and Refine), as declared in the document “Recognition and Alleviation of Pain in Laboratory Animals,” edited by the National Research Council (National Research Council (Us) Committee on Recognition, and Alleviation of Pain in Laboratory Animals., 2010).

Preliminary *in vitro* tests are usually performed before proceeding to *in vivo* tests on different animal models. For short gaps up to 15 mm mice and rats can be used; for longer defects, experimental models such as rabbit, pig, sheep, cat, dog or primate are generally adopted (Angius et al., 2012; Kornfeld et al., 2018). Most of *in vivo* models used to study biomaterials for NGCs are rats since this model is a compromise between mice, which allow performing nerve length-limited injuries, and larger animal models, the maintenance of which is more demanding and expensive, thus suggesting that they are more useful to test biomaterials in an advanced stage of NGC development. Indeed, if *in vivo* testing on rat fails, it is not recommended to test NGCs on larger animals.

*In vivo*, biomaterials are used as nerve guidance conduit (tubular conduit) or as conduit internal filler. *In vivo* tests allow to quantitatively assess nerve regeneration (i.e., myelin thickness, axon density and number, g-*ratio* and so on), functional recovery, evaluation of target re-innervation (Navarro, 2016), interactions between biomaterials and surrounding environment or interaction with re-growing tissues. *In vivo* tests must be performed to confirm the biocompatibility of the biomaterial, the non-toxicity of its degradation products, the scar formation and the absence of adverse immune response. Indeed, through *in vitro* assays, tissue reaction cannot be evaluated, in terms of local and systemic responses, because vascularization, extracellular matrix formation and oxygen supply are missing. Moreover, *in vivo* studies allow to evaluate long-term effects on the biomaterial under biological conditions: before clinical trial design, tests to evaluate the implanted biomaterial degradation rate must be carried out. When a device overcomes all preclinical tests, it is ready to be included in a clinical trial. Nevertheless, it is important to remember that animal models represent an approximation of human physiological and pathological processes (Talac et al., 2004; Angius et al., 2012; Kaplan et al., 2015). Moreover, it should also be kept in mind that not always clinical trials reach their completion or lead to satisfactory results. On the contrary, most of them fail during recruitment phases (stringency of inclusion/exclusion criteria) or in the follow-up phases (Carvalho et al., 2019). Thus, the commercial release of a device is a long-lasting, tiresome and expensive process. It is also important to remember that, beyond the research costs, biomaterial production and its release have costs; therefore, devices are often expensive and, for this reason, they have a reduced utilization in clinical practice.

In this review, the main characteristics of the ideal nerve conduit are summarized and discussed and the strengths and weaknesses of natural-based biomaterials used for nerve conduit manufacturing are reported. In particular, the description of preclinical outcomes of nerve regeneration after injury repair with natural-based conduits and the interactions between the biomaterial and regenerating tissue are the aim of this review.

## Biomaterial Characteristics to Be Used as a Nerve Conduit

**FIGURE 1:**
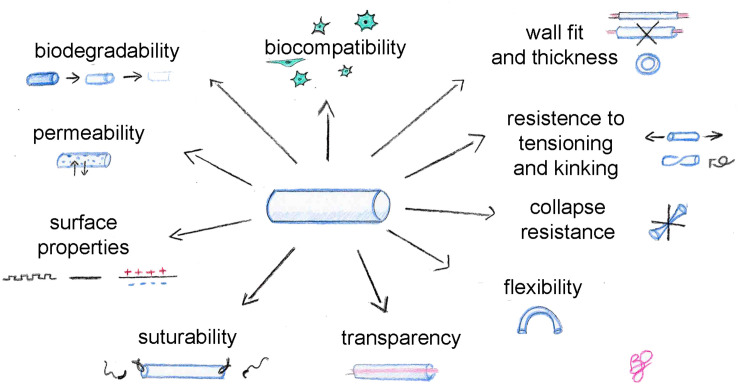
Characteristics to consider to obtain an ideal nerve conduit.

An ideal biomaterial, suitable for tissue engineering but also for nerve conduit production, must satisfy some requirements and find the right balance between different properties (Figure 1), such as biocompatibility, biodegradability, permeability, adequate biomechanical and surface properties. In addition, these specific physical features are recommended for conduits: flexibility, resistance to collapse and resistance to tension, adequate wall thickness, specific conduit diameter, and suturability. Transparency is not necessary, but it is appreciated by clinicians during surgeries.

Biocompatibility is assessed taking into account three parameters: (i) blood compatibility: the biomaterial in contact with the blood must not induce hemolysis or coagulation which can lead to thrombi formation; (ii) histocompatibility: the biomaterial must not induce side effects on the surrounding tissues, including scar formation, inflammation and any immune system response; (iii) mechanical compatibility: the biomaterial must present mechanical properties similar to the host tissue to avoid a local increase of tissue, reducing tissues natural/physiological mobility (Gu et al., 2011; Pinho et al., 2016).

Since passive mechanical forces influence the neuronal healing processes, it must be considered that a defined amount of mechanical stimulus helps nerve regeneration (Pfister et al., 2006; Loverde et al., 2011; Loverde and Pfister, 2015), but an exaggerated stimulus has opposite effects. In particular, strain forces transferred by the inner layer of the NGCs to the neuronal cell membranes must be lower than a certain level of strain considering that the mean strain resistance of cell membranes of sensory neurons [∼3,000 Pascal ([Pa)] and motor neurons (∼500 Pa) are different (Koch et al., 2012; Spedden et al., 2012; Moore and Staii, 2018) and that a higher mechanical force can damage the cell membranes activating cell apoptosis pathways. Also, the outer layer must mimic the resistance of the connective structures of the peripheral nerves which have a mean resistance of 580,000 Pa (Borschel et al., 2003; Giannessi et al., 2019).

The degradation kinetics of the substrate must be compatible with the regeneration timing of the specific tissue for which it is used. Ideally, the scaffold should be integrated into the surrounding tissue and gradually be replaced by the extracellular matrix and by the cells to restore the functionality of the tissue (Bačáková et al., 2004; Deng et al., 2008).

Conduit degradation should accommodate nerve regeneration timing: slow enough to maintain its strength and shape to guide and support axonal growth and not too quick to allow scar tissue formation around the re-growing axons between the two stumps (Chrząszcz et al., 2018; Houshyar et al., 2019). For example, too fast degradation of the conduit can cause an inflammatory response inducing a suboptimal nerve regeneration (Yu et al., 2011). Ideally, conduits should be fully resorbed when nerve repair is completed. Thus, the ideal degradation for a conduit should be faster at the proximal stump and gradually decreasing over distance with a slow degradation rate near the distal end (Reid et al., 2013).

Optimal degradation timing depends on defect length: longer nerve gaps require biomaterials with a longer degradation time, such as poly 3-hydroxybutyric acid (PHB), which is particularly appropriate for long-gap nerve injury repair. For a nerve gap of 10 mm, the axonal phase of nerve regeneration occurs around the third week of regeneration (Belkas et al., 2004; Carvalho et al., 2019). So, the conduit should ideally be significantly degraded after this phase, to mitigate entrapment-like symptoms and to abolish secondary surgeries required to remove the conduit, both are conditions commonly observed by using non-biodegradable conduits. Moreover, an ideal conduit should be semipermeable. Permeability is necessary for cell viability as it promotes the gas and nutrients exchanges and waste removal consisting of metabolites produced by the cells themselves (Ijkema-Paassen et al., 2004; de Ruiter et al., 2009; Liu and Hsu, 2020). Thus, an ideal scaffold for biomedical applications must supply a sufficient permeability, which also influences the ability to form fibrin matrices, useful during tissue regeneration processes to guide axons regeneration (She et al., 2008; Gu et al., 2011). Indeed, it has been shown that during the early stage of nerve regeneration, nerve wall permeability increases, and a similar behavior should be mimicked by the conduit (Rodríguez et al., 1999).

Conduit permeability increases with pore size (O’Brien et al., 2005): nerve conduits with large pores better support axonal growth in comparison with those displaying smaller pores (Clements et al., 2016; Liu et al., 2018). The optimal pore size range was 10–20 μm (Goulart et al., 2016), nevertheless, it should be higher than 4 μm and lower than 30 μm, thus allowing nutrients inflow and at the same time preventing extracellular matrix fibroblast incoming and growth factor outflow (Du and Jia, 2019).

In regards fibroblast ingrowth in the conduit during regeneration, it is needed to consider that different fibroblast types colonize the conduit: external fibroblast influx needs to be avoided, while nerve fibroblast migration from the two nerve stumps seems to have a positive role, as suggested by our recent paper (Fornasari et al., 2020). Nevertheless, the suggested 10–20 μm diameter seems too high, as it is known that cells can pass through smaller diameter pores (as shown in the Transwell migration assay, where cells pass through 8 μm pores), thus suggesting that probably <8 μm would be a better choice to allow nutrient exchange without undesired cell migration. An adequate permeability is important also to guarantee an adequate neurotrophic factor inflow inside the conduit, which is necessary for longer conduits since one common problem is the lack of neurotrophic factors required to support nerve regeneration. These characteristics should be tuned through conduit production methods.

A semipermeable nerve conduit is more effective to promote nerve regeneration when compared with low permeable or impermeable conduits (Rodríguez et al., 1999; Vleggeert-Lankamp et al., 2007; Dodla and Bellamkonda, 2008; Chiono and Tonda-Turo, 2015). Moreover, permeability is influenced by the hydrophilic properties of the material; moderate hydrophilicity of the material allows a better cell adhesion, compared to very high hydrophilicity or hydrophobicity (Liu et al., 2007).

Porosity affects the biomechanical/physical properties of the biomaterial, such as flexibility, resistance to collapse and tension, which are very important features considering the different biological and mechanical insults to which the conduit is subjected after *in vivo* implantation (Belkas et al., 2005). An adequate balance between conduit flexibility and stiffness should be achieved, as flexibility is a crucial requirement in clinical practice (de Ruiter et al., 2009; Gu et al., 2011). Indeed, digital nerve defects are the most frequent nerve injuries and flexible tubes are required for joint movements (Rasappan et al., 2018). Nevertheless, a certain mechanical resistance is required to avoid conduit collapse which could compromise nerve regeneration, since longer conduits are more subject to collapse or kinking. The collapse and/or kinking of the implant might compromise nerve regeneration too, by leading to nerve compression and ischemia (Pawelec et al., 2019).

Flexibility allows avoiding mechanical injuries of the surrounding tissues and the regenerating axons. Moreover, high rigidity of the implant may lead to nerve stump escape from the tube lumen and complicate the suturing processes of the device during implantation, indeed a not excessive rigidity allows an easy suturing procedure. Furthermore, the conduit should be resistant to tension to avoid tears from nerve tensioning during movements.

Surface properties, including surface functionalization, are not fundamental but should also be taken into account to enhance conduit performance and are important for the interaction between scaffold and cells (Ratner and Bryant, 2004). Surface chemistry alteration is an effective strategy to promote cell adhesion and neurite outgrowth (Oh et al., 2013; Sarker et al., 2018).

A large surface area promotes cell adhesion and proliferation, an oriented surface influences cell behavior (Ahmed et al., 2003; Jiang et al., 2010). Macro (>10 μm), micro (between 0.5 and 10 μm) and nano (<0.5 μm) roughness can determine a different adhesion pattern (Rajab et al., 2017; Carvalho et al., 2019). Nanostructured conduit internal surfaces could improve cell adhesion, while microstructured surfaces are useful to target regrowing axons toward the target organ. Thus, an internal microstructured surface could be useful for conduits to repair long defects, where neurotrophic factor diffusion is not enough to reach regrowing axons (Wang et al., 2001; Sun and Downes, 2009). The conduit surface is generally modified from smooth to rough in three ways: by adding grooves, holes or raised parts (Sun and Downes, 2009; Tonda-Turo et al., 2011a; Gnavi et al., 2015). Differences in nanoroughness of the same biomaterial can affect the NGC wettability (Yang et al., 2005; Sun and Downes, 2009); moreover, nanometric roughness positively influences cell adhesion as it mimics the extracellular matrix structure (Marshall et al., 2010) and enhances cell growth and cytoskeleton elongation (Huang et al., 2015). Microstructured scaffolds might be useful to mimic bigger structures, such as the bands of Büngner, encouraging axonal regrowth during peripheral nerve regeneration (Bozkurt et al., 2012; Gnavi et al., 2015). Thanks to several manufacturing processes like laser surface texturing, chemical substances, plasma treatment, electrospinning or use of a mold, changes in roughness can be made for most of the available biomaterials (Kim et al., 2016; Berkovitch et al., 2018; Chen et al., 2019).

Furthermore, surface charges can enhance the biological response *in vivo*. The electrical charge or conductivity of a material influences cell colonization; cells adhere to negatively charged substrates because of the presence of positive charges on the cell membrane surface (Lesný et al., 2006; Bacakova et al., 2011). Moreover, neurite outgrowth can be increased by electrically conducting polymers (Schmidt et al., 1997; Dadsetan et al., 2009; Jing et al., 2018).

Nerve regeneration outcome is also influenced by the nerve guide diameter, which has to match with the dimension of the proximal and distal stumps of the injured nerve. Conduits not fitting well with the nerve stumps might compromise nerve regeneration: smaller diameters might lead to chronic nerve compression, while a conduit with too large diameter allows the incoming of undesired cells and fibrous tissue formation, in addition to adverse growth factors outflow (Daly et al., 2012; Isaacs et al., 2014; Yi et al., 2019). Thanks to the physical and chemical properties of the biomaterials described, internal conduits diameter can be quite easily modified through conduit processing methods.

Also, the wall thickness must be considered during nerve guide design, because as reported by Ducker and Hayes there is a strong correlation between conduit wall thickness and neuroma formation (Ducker and Hayes, 1968). Wall thickness influences different characteristics such as permeability, but also mechanical properties like the flexibility and solidity of the conduit, thus it should be considered during the conduit design processes.

Conduit walls more than 0.8 mm thick reduce axonal growth (Rutkowski and Heath, 2002), and this reduction is attributed to permeability and porosity reduction, which are important for nerve regeneration. Indeed, *in silico* experiments suggest that a 0.6 mm wall thickness, with an 80% porosity, can be considered optimal for nerve regeneration (Kokai et al., 2009; Tonda-Turo et al., 2011a; Chiono and Tonda-Turo, 2015). Data obtained from *in vitro* and *in vivo* experiments on rat sciatic nerve injury model demonstrated that a wall thickness lower than 0.6 mm or higher than 0.8 mm is suboptimal and leads to controversial results (Rutkowski and Heath, 2002; Stang et al., 2009; Tonda-Turo et al., 2011a; Chiono and Tonda-Turo, 2015; Teuschl et al., 2015; Wang et al., 2017; Jahromi et al., 2020). Nevertheless, further studies are necessary to identify the more adequate thickness, since in literature conflictual *in vivo* results were published.

Wall thickness is also important since it is one of the parameters influencing conduit suturability. An ideal conduit could be easy to suture; the nerve guides must allow the suture needle to pass through the wall avoiding the escaping of the nerve stumps from the conduit lumen, but also they must be strong enough to allow the suture to bind the proximal and distal nerve stumps without detaching if subjected to tension forces during movement (Nectow et al., 2012).

Finally, transparency is a characteristic appreciated by surgeons because it allows the optimal positioning of nerve stump ends during nerve repair surgery. This characteristic is also useful during preclinical assays since it allows to directly observe *in situ* if nerve regeneration occurs (Gu et al., 2014).

## Natural-Based vs. Synthetic Biomaterials

Biomaterials of natural origin are suitable for tissue engineering as they provide adhesion molecules, cell binding sites and are compatible with surrounding tissues. Because of their large availability, large quantities of natural-based biomaterials are accessible at reasonable prices (Dalamagkas et al., 2016), but their purification is less standardized in comparison to synthetic biomaterials. Thus, the problem with such natural biomaterials is the difficulty to find an easily accessible purified source for large-scale production. Nevertheless, some biopolymers overcome this problem, such as PHB, which is synthesized by bacteria in bioreactors (Koller, 2018), and chitosan, which is extracted from chitin, an abundant polysaccharide derived from shellfish waste. Indeed, it has been estimated that more than 10,000 tons are produced by shellfish waste each year: quantities, which could provide enough material to cover market demands for tissue engineering (Hamed et al., 2016).

Natural biomaterial advantages derive from the fact that, after a proper purification, these biomaterials generally do not determine unexpected or unwelcome immune-mediated responses. They provide better biocompatibility in comparison to synthetic ones: usually, these biomaterials are biodegradable and integrate with the surrounding tissues. Their degradation products, compared to synthetic ones, are less cytotoxic and more biocompatible as well as more easily degraded and metabolized by the host tissues (Stoppel et al., 2015).

Natural polymers, thanks to their excellent biocompatibility and bioactive properties, allow better interactions between the scaffold and the tissue, which improve cell adhesion, proliferation and tissue regeneration (Arslantunali et al., 2014; de Queiroz Antonino et al., 2017).

However, natural-based biomaterials present some limitations, such as the need for extensive purification or chemical heterogeneity, which leads to variable mechanical properties like degradation rate. Besides, natural polymers usually possess poor mechanical properties and batch-to-batch variability which have restricted their widespread use (Schmidt and Leach, 2003).

On the other hand, synthetic biomaterials own more tunable mechanical properties, which can be obtained through small changes during the manufacturing process (Dalamagkas et al., 2016). This characteristic, which determines synthetic biomaterial high reproducibility, together with the easier access to large-scale production, makes them an appealing source. For many years the lack of binding sites and the reduced biocompatibility of synthetic biomaterials were great concerns; anyhow, currently, tissue engineering goes beyond the problem through the design of scaffolds enriched with nanostructured surface topography, which offer binding sites to cells (Singh et al., 2014). Scaffold surface topographical modifications influence cell growth, migration and adhesion by affecting actin cytoskeleton reorganization, focal adhesion formation and distribution and *lamellipodia* and *filopodia* formation (Mattila and Lappalainen, 2008; Harvey et al., 2013; Rahmany and Van Dyke, 2013; Zhao et al., 2020). Moreover, it has been demonstrated that topographic cues improve axonal growth and could minimize atrophy of innervated organ distal to the lesion resulting in nerve functional recovery (Chiono and Tonda-Turo, 2015).

## Natural-Based Biomaterials

The most studied natural-based biomaterials used to support nerve regeneration (Table 1) are polysaccharides (hyaluronic acid, alginate, chitin and chitosan), proteins (collagen, gelatin, silk fibroin, fibrin, and keratin) and polyesters derived from natural sources [poly (3-hydroxybutyric acid) and poly (3-hydroxybutyric acid-co-3-hydroxyvaleric acid)] (Arslantunali et al., 2014; Raza et al., 2020). Nevertheless, hyaluronic acid, alginate and keratin, discussed in the following paragraphs, do not possess enough mechanical strength to be used alone to produce a NGC, but can be used as internal fillers for nerve conduits with successful results.

**TABLE 1:** Advantages and disadvantages of natural-based biomaterials used as nerve conduits.

Natural-based biomaterial	Advantages	Disadvantages
Hyaluronic acid	- Producible in large scale by microbial fermentation (Hemshekhar et al., 2016). - Supports axonal regrowth (Wang et al., 1998; Zhang et al., 2008). - Successfully used as conduit internal fillers (Xu et al., 2017).	- Very low mechanical properties (too weak to manage for producing a conduit) (Wang et al., 1998; Zhang et al., 2008). - Fast degradation (Drury and Mooney, 2003; Jansen et al., 2004).

Alginate	- Remarkable chemical flexibility (Kim and Kim, 2014). - Usable blended with other polymers (Kim and Kim, 2014). - Manufactured with several techniques (electrospinning, 3D printing, …) (Sergeeva et al., 2019; Singh et al., 2020). - Usable as a conduit internal filler (Pfister et al., 2008).	- Weak mechanical resistance for using as a conduit if not blended with other polymers (Hashimoto et al., 2002). - High degradation rate (Golafshan et al., 2017).

Chitin and chitosan	- Chitin: the most abundant polysaccharide, after cellulose, in nature (Martínez et al., 2014). - Chitosan: easily obtained from chitin, at low cost (Freier et al., 2005a). - Chitosan: supports axonal regrowth and reduces scar formation (Haastert-Talini et al., 2013; Stenberg et al., 2016; Neubrech et al., 2018). - Degradation products positively influence nerve regeneration (Gong et al., 2009; Zhao et al., 2017). - Different strategies to increase its mechanical strength: double layered conduits (Wang et al., 2008a, b) or biomaterial blending (Fan et al., 2008; Boecker et al., 2019). - Transparency, flexibility and resistance to collapse (Neubrech et al., 2016, 2018).	- Low mechanical strength (Boecker et al., 2019).

Collagen	- The most abundant protein in the human body (Pabari et al., 2010). - Very good vehicle for drug and cell delivery (Dalamagkas et al., 2016). - Its physical features along different parts of the same conduit can be modified (Aigner et al., 2020). - Topographical cues that guide the axon regrowth can be obtained (Stang et al., 2005a, b).	- Low grade of resistance to mechanical stress and weak manipulability (Schmidt et al., 2011). - Some scar neuroma cases in patients (Liodaki et al., 2013; Rbia et al., 2019).

Gelatin	- Plasticity, adhesiveness and low antigenicity (Lien et al., 2009). - Bioactive molecules can be easily incorporated in the gelatin scaffolds and gradually released during its degradation, thanks to its chemical properties and degradation kinetics (Chen P. R. et al., 2005).	- Solubility in water and the easy collapse of gelatin conduits make necessary the use of various cross-linking agents (Liu et al., 2004; Tonda-Turo et al., 2011b).

Silk fibroin	- Economically advantageous because of its easy and cheap purification processes and its large-scale availability (Kundu et al., 2013). - Good elasticity, flexibility, and high resistance to fracture and compression (Omenetto and Kaplan, 2010). - Degradation influenced by processing methods (Wang et al., 2008c; Li et al., 2019). - No signs of adverse immune response (Yang et al., 2007b). - Good mechanical strength (Yang et al., 2007b).	- Silk fibroin solutions are generally weak and fragile (Kundu et al., 2013).

Fibrin	- Adhesive characteristics (Park and Woo, 2018; Wang et al., 2018b). - No collapse (Takazawa et al., 2005; McGrath et al., 2018). - Enough porosity to allow nutrient inflow (Takazawa et al., 2005; McGrath et al., 2018). - Prevents fibrous tissue formation (Takazawa et al., 2005; McGrath et al., 2018).	- High degradation rate (Rowe et al., 2007). - Conduits made by autologous fibrin are more effective than commercial ones (De la Puente and Ludeña, 2014).

Keratin	- Biodegradable, bioactive and with a hydrophilic surface (Rajabi et al., 2020). - Good as conduit filler (Apel et al., 2008; Pace et al., 2014). - Usable blended with other polymers (Gupta and Nayak, 2016).	- Weak mechanical resistance for using as a conduit (Rajabi et al., 2020). - Fast degradation rate (Rajabi et al., 2020).

PHB	- Stable local pH during degradation (Koller, 2018). - Good mechanical and physical features, modifiable by different production methods (Masaeli et al., 2012). - Early vascularization after implantation (Mohanna et al., 2005). - Low levels of inflammatory infiltration (Hazari et al., 1999).	- Long reabsorption time (over 2 years) (Mohanna et al., 2005). - Tendency to crystallization leading to reduced flexibility and ductility (Zhuikov et al., 2020). - Fragility and lack of hydrophilicity (Zhuikov et al., 2020).

PHBV	- More flexible and easier to process than PHB (Zhuikov et al., 2020). - Easy to handle and to suture (Biazar et al., 2013a).	- A narrow processing window and a low strain-at-break in comparison to petroleum-based synthetic polymers (Zhao and Turng, 2015).

### Polysaccharides

#### Hyaluronic Acid

Hyaluronic acid (HA) is a glycosaminoglycan which composes the extracellular matrix and, interacting with other extracellular molecules, is involved in the regulation of different cellular processes (Hemshekhar et al., 2016). To obtain a large amount of HA and to avoid animal-derived pathogen risks, HA could be produced in large-scale by microbial fermentation. Mechanical properties and degradation of HA can be tuned through chemical and physical processes of crosslinking with divinyl sulfone followed by freezing and lyophilization to create a porous structure (Ortuno-Lizarán et al., 2016; Vilariño-Feltrer et al., 2016). Moreover, HA can be solved with sodium chloride and directly poured into a porous sponge (Li et al., 2018; Entekhabi et al., 2020) or suspended in physiological saline solution to obtain a suitable viscosity useful to produce hydrogel fillers for NGCs (Li et al., 2018).

Hyaluronic acid is characterized by non-adhesive cue, is biocompatible and supports axonal regrowth (Wang et al., 1998; Zhang et al., 2008), but it owns very low mechanical properties. Indeed, even if blended with other biomaterials such as chitosan, it is too weak to manage (Li et al., 2018). *In vivo* it is degraded by hyaluronidases, which are widely diffuse in the organism, determining its fast degradation (Drury and Mooney, 2003).

It has also been reported that a nerve conduit based on an esterified HA derivative showed a fast degradation, not compatible with the timing necessary to support nerve regeneration, and the formation of fibrous tissue and a substantial cell ingrowth was observed (Jansen et al., 2004).

These characteristics make HA unsuitable as a conduit for nerve regeneration, but for its characteristics is very suitable as conduit internal filler, mostly in hydrogel form (Xu et al., 2017).

#### Alginate

Alginate is commonly extracted from brown seaweed and thanks to its biocompatibility is widely used in biomedical applications (Shen et al., 2005). The alginate is composed of mannuronic acid and guluronic acid which confer remarkable chemical flexibility compared to other degradable biocompatible materials with a notable resemblance to the mammalian extracellular matrix structure (Kim and Kim, 2014). It is easily modifiable via chemical reactions [e.g., alginate dialdehyde formed by periodate oxidation of sodium alginate) (Dranseikiene et al., 2020) and physical crosslinking using Ca ions, maintaining a negligible inflammatory response (Sun and Tan, 2013)].

This biomaterial can promote nerve regeneration but has a weak mechanical resistance, insufficient to bear physiological loading conditions and the high degradation rate justifies the use of alginate blended with other polymers (Hashimoto et al., 2002; Omidian et al., 2006; Kim and Kim, 2014; Shen and Hsieh, 2014; Golafshan et al., 2017), hybridized by incorporating nanofillers (Homaeigohar et al., 2019) or both (Chen et al., 2019). Alginate blending with biomaterials of natural origins could be a successful strategy: the research performed by Pfister et al. (2007a) demonstrated the effectiveness of a blend conduit consisting of alginate and chitosan to support nerve regeneration for short nerve gaps. This conduit possesses a good permeability and an adequate mechanical strength, which also allows an easy suturing, and promotes cell adhesion thanks to its hydrophilic nature (Pfister et al., 2007a).

The physical properties of alginate make it suitable to be manufactured with several techniques such as magnetic templating (Singh et al., 2020), electrospinning (Kim and Kim, 2014; Chen et al., 2018; Kuznetsov et al., 2018), microfluidics (Costantini et al., 2016; Hu Y. et al., 2017), gas foaming, emulsion freeze drying, 3D printing (You et al., 2017), and hard templating on vaterite CaCO_3_ crystals (Sergeeva et al., 2019).

Alginate is also used successfully as a conduit internal filler for growth factor delivery in nerve regeneration (Ohta et al., 2004; Pfister et al., 2007b, 2008) and for molecule controlled release at various pH values (Augst et al., 2006; Gao et al., 2009; Jeon et al., 2009). In particular, alginate as internal filler leaves no residue 4 months after surgery in a cat model of 50 mm gap sciatic nerve injury repaired with tubulation (Sufan et al., 2001) and is also able to promote specifically adhesion and proliferation of nerve cells in rats with 10 mm sciatic nerve gap 7 weeks after surgery (Askarzadeh et al., 2020).

#### Chitin and Chitosan

Chitin is a linear homopolymer of *N*-acetyl-D-glucosamine, belonging to the glycosaminoglycan family; in nature, it is the most abundant polysaccharide, after cellulose, and it is obtained from arthropod exoskeleton (Martínez et al., 2014).

Crustacean shells, due to their abundance, are considered the main source for chitin isolation; indeed, chitin derived from their wastes represents around half of shellfish total weight. Chitin can be extracted with biological (microbial) or chemical methods (Ramírez et al., 2010; Hamed et al., 2016).

Chitin is mainly used in its partial deacetylated form, chitosan, in many different fields ranging from the food industry and agriculture (Boonlertnirun et al., 2008) through pharmaceutics (Varshosaz, 2006; Mitsou et al., 2020) to regenerative medicine (e.g., biomedical patches, artificial skin and orthopedic tissue engineering, nerve conduits) (Shapira et al., 2016; Porpiglia et al., 2018; Sandri et al., 2019; Ryu et al., 2020; Tao et al., 2020a). Chitosan can be easily obtained from chitin, at low cost, through alkaline hydrolysis (Freier et al., 2005a).

Chitosan is widely used to support peripheral nerve regeneration and its effectiveness has been investigated in several studies (Table 2). It possesses many suitable characteristics to be used in this field: it is biocompatible (Yuan et al., 2004; Freier et al., 2005a; Simões et al., 2011), it can support axon regrowth (Haastert-Talini et al., 2013; Stenberg et al., 2016) and reduce scar tissue formation (Neubrech et al., 2018). Moreover, it is a re-absorbable biomaterial whose degradation products (including chito-oligosaccharides) positively influence nerve regeneration (Gong et al., 2009; Zhao et al., 2017). Furthermore, chitosan can be processed through many fabrication technologies (Ruini et al., 2015; Tonda-Turo et al., 2017b) to produce NGCs both as external wall material and cell-based therapies as internal filler (Boido et al., 2019; Tonda-Turo et al., 2020). One factor limiting chitosan use as a nerve guide is its low mechanical strength; nevertheless, Freier et al. (2005b) demonstrated that modifying the chitosan acetylation degree, its mechanical stability can be improved and conduits with different acetylation degree were also tested *in vivo* (Haastert-Talini et al., 2013). Other strategies can be used either to increase chitosan mechanical strength, either to avoid the guide collapse, such as double layered conduit production (Wang et al., 2008a, b) or chitosan blending with biomaterials presenting a high mechanical force (Fan et al., 2008; Boecker et al., 2019). Indeed, chitosan has been extensively studied *in vivo*, in combination with different synthetic polymers, successfully bridging nerve defects (Jiao et al., 2009; Ding et al., 2010). For example, Xie et al. (2008) tested with positive results, a chitosan-polylactic acid (PLA) blend, taking advantage of PLA mechanical properties and of chitosan biocompatibility.

**TABLE 2:** Relevant studies on chitosan based-conduits.

	References	Method of conduit production	Analyses	Results
**Chitosan**	Haastert-Talini et al., 2013	Extrusion process (Medovent GmbH, Mainz, Germany), followed by washing and hydrolysis steps to obtain different degrees of acetylation (DA). DAI 2%; DAII 5%; DAIII 20%.	- *In vitro* analysis. - Analysis on 10 mm rat sciatic nerve gap repaired with chitosan conduit for short and long-term analysis. - Morphological analysis at short times: connective tissue assessment, regenerated matrix analysis inside the conduit and IHC. - Functional recovery evaluation: static sciatic index (SSI) and electrophysiology. - Long-term analysis (13 weeks): morphometrical assessment of the regenerated nerve tissue, stereological and conduit analysis.	- No *in vitro* toxicity. - Short term: higher number of activated Schwann cells in the distal segments of nerves regenerated through DAIII tubes. - Chitosan tubes with the different DAs allowed good structural and functional sciatic nerve regeneration. - Differences with regard to the speed of their degradation: DAI no degradation after 3 months, DAIII faster degradation. - DAIII conduit showed a lower mechanical stability in comparison with the other experimental groups. - Nerves regenerated through DAI chitosan tubes revealed a significantly higher total number of myelinated axons as compared to the gold standard. - SSI and electrophysiology showed no differences between the experimental groups. - No differences in connective tissue thickness.
	
	Shapira et al., 2016	Extrusion process (Medovent GmbH, Mainz, Germany)	- 10 mm rat sciatic nerve gap was repaired for 3 months. - Morphological (ultrasound imaging, macroscopical evaluation and muscle weight assessment) and morphometrical analysis. - Electrophysiology and sciatic functional index (SFI) evaluation.	- Ultrasonography showed no conduit detachments or collapses. - Muscle weight assessment, histomorphometry, functional and electrophysiological outcomes were similar between the chitosan tube and autologous nerve graft group. - No differences between the experimental groups were detected for morphometrical analysis (total number of myelinated fibers, fibers diameter, myelin thickness and *g-ratio*).
	
	Gonzalez-Perez et al., 2015	Extrusion process (Medovent GmbH, Mainz, Germany), followed by washing and hydrolysis steps to obtain different degree of acetylation (DA). DAI 2%; DAII 5%	- Analysis on rat sciatic nerve gap of 15 mm, repaired with chitosan conduit for short and long- term analysis (7, 30, 60, 90, and 120 days). - Morphological and morphometrical analysis. - Functional Evaluation of Sensory Recovery (Von Frey test) and electrophysiology. - Muscle weight assessment.	- Earlier and higher muscle reinnervation in rats repaired with autograft in comparison with chitosan groups. - A larger and higher number of myelinated fibers was observed in the autograft group in comparison to chitosan experimental group. - Similar mean size of the myelinated fibers in chitosan and autograft groups was observed.
	
	Yin et al., 2018	Chitosan conduit obtained from a freeze-cast process.	- 10 mm sciatic nerve gap was repaired for 12 weeks with a porous chitosan conduit or through autograft technique. - Morphological analysis (Hematoxylin and eosin and IHC).	- Axonal outgrowth across the conduit was observed. - Conduit porosity allowed a good angiogenesis and prevented scar formation.
	
	Ao et al., 2011	Chitosan conduit obtained from a mold-mandrel processing.	- Characterization of morphology and mechanical properties of chitosan conduit. - 12 mm rat sciatic nerve gap was repaired with empty or cell-enriched chitosan conduit for 3 months. - Electrophysiology SFI evaluation. - Muscle weight assessment. - Morphological analysis (histological stainings and IHC).	- After 3 months the conduit became thinner but still maintained its lumen and wall integrity. - Similar recovery in terms of myelinated axon number and conduction velocity in all experimental groups was observed.

Chitosan is an attractive material for nerve guide manufacturing because of its versatility, and its surface texture can also be easily modified to better support axonal regrowth (Shuai et al., 2013; Wrobel et al., 2014).

In recent years, various studies on chitosan-made tubes have demonstrated chitosan conduit efficacy in inducing nerve regeneration for bridging peripheral nerve defects of 8 mm or more in rat models (Ao et al., 2011; Haastert-Talini et al., 2013; Gonzalez-Perez et al., 2015; Shapira et al., 2016; Ronchi et al., 2018; Yin et al., 2018; Crosio et al., 2019). 8 mm median nerve defects were also immediately or delayed repaired with chitosan conduits to test if chitosan guide enrichment with muscle fibers, used as internal filler, better promote nerve regeneration; morphometrical and functional analysis demonstrated that nerve regeneration was obtained in both experimental groups with similar results, suggesting that for short gaps the use of hollow chitosan guide is sufficient to obtain nerve regeneration (Ronchi et al., 2018; Crosio et al., 2019). Another study investigated nerve regeneration in rat sciatic nerves 3 months after 10 mm nerve repair with chitosan conduits with three different deacetylation degrees (Haastert-Talini et al., 2013) or with autografts, showing no significant differences among all the experimental groups at functional, biomolecular and morphological levels. Similar results were obtained by Shapira et al. (2016) which observed, 3 months after 10 mm nerve gap repair, that electrophysiological, morphological, morphometrical outcomes were comparable between nerve repair through autograft or chitosan tube.

Similar experimental conditions (10 mm gap and 3 months post-operative) were used to test nerve regeneration inside a freeze-cast, double-layered chitosan tube, where authors observed that the conduit porosity allows good angiogenesis and prevents scar formation. Nevertheless, this study needs further analysis to be completed (Yin et al., 2018).

Experimental studies of nerve gaps longer than 10 mm repaired with chitosan tubes showed that chitosan conduit permits nerve regeneration, even if lower than those of nerve repaired through the autologous graft. Gonzalez-Perez et al. (2015) tested chitosan tubes with two different deacetylation levels on 15 mm nerve defects; 4 months after the repair they obtained a similar number of myelinated fibers and similar functional and electrophysiological results. As additional experimental group, they tested nerve regeneration in a silicon tube where nerve regeneration was not observed. Also, Ao et al. (2011) observed for the critical nerve gap length of 12 mm nerve regeneration inside a hollow chitosan conduit, although only cell enriched experimental groups showed morphological and functional results similar to those achieved by the autograft group.

Different clinical trials on chitosan based-conduits were planned during these years, and in 2015 a chitosan nerve conduit, Reaxon^®^, was commercialized; it allows to bridge nerve gaps up to 26 mm. Transparency, flexibility and resistance to collapse are amongst the advantages of the Reaxon^®^ conduit. In clinical practice, it has been shown that the use of this conduit for hand nerve injury repair with end-to-end technique positively influences sensory and motor recovery in patients treated with this conduit (Neubrech et al., 2016, 2018). In order to optimize the regeneration process, manufacturers suggest using the Reaxon^®^ Nerve Guide in anatomical sites able to maintain it moistened and to avoid its drying and its consequent stiffening.

### Proteins

#### Collagen

The main reason why collagen has been so commonly used to create nerve conduits in the last three decades (Eppley and Delfino, 1988; Pabari et al., 2010) is that it is the most abundant protein in the human body (Pabari et al., 2010). Collagen is a major component of the extracellular matrix and of the peripheral nervous system envelope. It can be isolated from many biological tissues (e.g., tendon, skin, and bone). Collagen is a very good vehicle for drug and cell delivery (Dalamagkas et al., 2016) and it is possible to modify its physical features along different parts of the same conduit (Aigner et al., 2020). The different binding domains on it give the possibility to create topographical cues that guide the axon regrowth (Stang et al., 2005a, b) facilitating cell adhesion, survival and migration (Alberti et al., 2014; Sulong et al., 2014; Drobnik et al., 2017a, b). Many researchers have shown that collagen has biological properties superior to other materials available in the market for nerve scaffold fabrication and it is also effective over a nerve gap distance of at least 15 mm (Archibald et al., 1991). 10 mm long hollow conduits reported better results in rat nerve regeneration and muscle re-innervation if compared to collagen polyglycolic acid (PGA)-filled conduits (Saltzman et al., 2019). A conduit made by mixing types I and III collagen filled with collagen filaments was not inferior to autologous graft in treating 30 mm nerve defects, with 80% of patients reporting sensory recovery after 12 months (Saeki et al., 2018). There are few collagen nerve conduits approved by the Food and Drug Administration (FDA) and Conformity Europe (CE). NeuraGen^®^, a nerve conduit made of type I bovine collagen derived from the Achilles tendon (Di Summa et al., 2014), reported overlapped results compared to allograft 12 months post-surgery in patients with the reconstruction of a digital nerve gap inferior to 2.5 cm (Rbia et al., 2019). However, some scar neuroma cases, with and without foreign bodies, imposed conduit removal (Liodaki et al., 2013; Rbia et al., 2019). Maybe due to the tube high stiffness and high cost, it is not so popular among surgeons (Arslantunali et al., 2014; Sedaghati et al., 2014). RevolNerv^®^, a porcine skin-derived collagen type I based-conduit, showed good clinical outcomes and the absence of post-surgical neuromas in 163 patients that underwent wrapping direct end-to-end sutures (Thomsen and Schlur, 2013) and comparable results with uncoated direct sutures for palmar digital nerves were observed (Arnaout et al., 2014). Neuroflex^®^ and NeuroMatrix^®^, respectively, made in collagen types I and III, were approved in 2001, but clinical and preclinical researches are still lacking (Meek and Coert, 2008; Stang et al., 2009; Chrząszcz et al., 2018). No clinical and preclinical researches have shown immune response due to allogenic origin of collagen NGCs (Archibald et al., 1995; Boeckstyns et al., 2013; Chrząszcz et al., 2018). Indeed, the high purification process by which those NGCs are manufactured is able to remove all impurities that can trigger an immune response (Wong et al., 2014; Maurer et al., 2018). Because collagen has such powerful clinical effects, but low grade of resistance to mechanical stress and weak manipulability (Schmidt et al., 2011), future researches will be necessary to tackle these problems. Indeed, collagen could also be blended with other biomaterials like silk fibroin or chitosan, that are able to increase dramatically its mechanical resistance (Huang et al., 2011; Teuschl et al., 2015; Xu et al., 2016).

#### Gelatin

Gelatin is obtained by the thermal denaturation of collagen. Animal-derived gelatin has been widely investigated for medical applications, for its biocompatibility, its plasticity and adhesiveness. Gelatin is less expensive than collagen and has relatively low antigenicity compared to its precursor (Lien et al., 2009). Nevertheless, its solubility in water and the easy collapse of gelatin conduits make necessary the use of various cross-linking agents, resulting in the alteration of its mechanical and physical properties with a controlled degradation rate (Liu et al., 2004; Tonda-Turo et al., 2011b) (Table 3).

**TABLE 3:** Relevant studies on protein based-conduits.

	References	Method of conduit Production	Analyses	Results
**Gelatin**	Chang et al., 2009	Genepin cross-linked gelatin solution poured into a mandrel	- A non-porous and a porous genepin cross-linked gelatin conduits were compared and used to repair a 10 mm rat sciatic nerve gap up to 12 weeks. - Macroscopical observation and characterization of the conduits (porosity, mechanical properties, swelling ratio, water contact angle analysis, and cytotoxicity assessment). - Electrophysiology. - Morphological and morphometrical analysis.	- Porous gelatin conduit showed a faster degradability and lower mechanical strength in comparison to the non-porous one. - Rats repaired with the porous conduit presented a significant higher nerve conductive velocity. - Mostly of the regenerated axons in the non-porous conduit group were unmyelinated. - Porosity implement gelatin conduit performance, with good nerve regeneration outcome.
	
	Liu, 2008	Proanthocyanidin cross-linked gelatin solution placed into a silicone tube used as inner mandrel.	- *In vitro* enzymatic degradation assays and biocompatibility assessment. - 10 mm rat sciatic nerve defect was repaired with proanthocyanidin cross-linked gelatin conduit for 8 weeks. - Macroscopical and microscopical observation of the conduit. - Electrophysiology and morphological analysis.	- Conduit has resisted to degradation by digestive enzymes. - Gelatin and proanthocyanidin release was observed and it seems to support Schwann cell adhesion and growth. - Conduit was well integrated in the surrounding tissues, holding its shape and no inflammatory reaction was observed 8 weeks after the repair. - Regenerated fibers contained high number of unmyelinated and myelinated axons.
	
	Gámez et al., 2004	Photo-fabrication of the gelatin conduit.	- 10 mm rat sciatic nerve gap was repaired with gelatin conduit up to 12 months. - Hollow conduit performances were compared with those of the same conduit enriched with fibers alone or impregnated with bioactive molecules. - Functional evaluation of motor recovery and electrophysiology assessment. - Macroscopical evaluation and morphological analysis (light microscopy and IHC).	- At 12 weeks the photocured gelatin conduit was degraded and adsorbed without signs of inflammatory reactions. - Morphological, functional, and electrophysiological response recovery was observed for all experimental groups. - Nerve regeneration was better in the two enriched experimental groups, in terms of number and maturity of regenerated axons, but also in terms of functional recovery.
	
	Ko et al., 2017	Bisvinylsulfonemethyl cross-linked gelatin solution poured into a mandrel.	- Conduit characterization (SEM, analysis of the tensile force and of water contact angle, biocompatibility and degradation). - 10 mm rat sciatic nerve defect was repaired with gelatin conduit up to 8 weeks and compared with silicone conduit. - Electrophysiology. - FluoroGold retrograde tracing. - Morphological (light microscopy, TEM and IHC) and morphometrical analysis. - Protein expression analysis.	- Biocompatible conduit. - Gelatin cross-linking with bisvinylsulfonemethyl reduced gelatin swelling and improved its mechanical properties. - Conductivity was observed in all rats, indicating that nerve fibers had successfully reinnervated. - Gelatin conduit induced lesser macrophage infiltration into the regenerated nerves in comparison to silicone conduit. - Expression of neuron-related growth factors, such as IGF-1, BDNF, and GDNF was observed.

**Silk fibroin**	Yang et al., 2007b	Freeze-drying procedure.	- Silk fibroin conduit with oriented filaments inside was used to repair a 10 mm long rat sciatic nerve defect for 6 months. - Conduit characterization with mechanical and permeability testing. - Electrophysiology. - FluoroGold retrograde tracing. - Morphological analysis (histological stainings, IHC and electron microscopy.	- Good mechanical and permeable properties. - No signs of adverse immune response. - Morphological and functional outcomes close to those obtained with autograft group.
	
	Park et al., 2015	Electrospinning technique.	- *In vitro* tests to evaluate conduit mechanical properties and cytotoxicity. - Conduit was used to repair a 10 mm rat sciatic nerve gap for 10 weeks and compared with autograft. - Evaluation of functional recovery (ankle stance angle test). - Morphological analysis (IHC).	- *In vitro* tests on Schwann cells showed conduit cytocompatibility. - Myelinated axonal fibers were observed. - A restore in motor function was observed 10 weeks after the repair.
	
	Belanger et al., 2018	Tri-layered silk conduit obtained through electrospinning technique.	- SEM, mechanical strength test, electrospun fiber diameter and angle evaluations. - *In vitro* assays (IHC) on Schwann cells. - Silk fibroin conduit was used to repair 5 mm rat sciatic nerve gap for 4 and 8 weeks in comparison with a direct suture. - Morphological analysis (IHC).	- Conduit with an optimized surface architecture and mechanical properties. - Conduit resistance to tearing. - 4 months after the injury, histological analysis revealed regenerated nerve fibers in both experimental groups. - Myelin thickness and axon diameter average at 8 months was similar in both experimental groups.
	
	Alessandrino et al., 2019	SilkBridge: a tri-layered silk conduit with a textile layer between two layers obtained through electrospinning technique.	- Morphological, physical, chemical, and mechanical scaffold characterization. - *In vitro* assays on Schwann cell and motor neuron cell lines. - *S*ilk fibroin conduit empty or filled with microfibers were used to repair a 10 mm median nerve gap for 2 and 4 weeks. - Macroscopical evaluation and morphological analysis (high resolution light microscopy, electron microscopy and IHC).	- Resistance to compression, desirable wall thickness and porosity values. - *In vitro* tests confirms scaffold biocompatibility. - No sign of inflammation or scar tissue formation were observed. - Conduit integration with the surrounding tissues and cell colonization was observed. - The progressive presence of some myelinated fibers at the proximal nerve stump was observed.

**Fibrin**	Kalbermatten et al., 2009	Fibrin glue was pulled into a specially designed compactor with a silicone inlay around a stainless steel core.	- Fibrin conduit effectiveness was compared with that of a PHB conduit to repair a 10 mm rat sciatic nerve gap for 2 and 4 weeks. - Morphological analysis (macroscopical evaluation and IHC).	- Fibrin conduit did not collapse. - 2 weeks after the repair a better axon regeneration length was observed in comparison to PHB conduit groups. - Full crossing of axons after 1 month was shown.
	
	McGrath et al., 2018	Fibrin glue components were mixed and a silicone mold with a central metal rod was used to prepare the conduit.	- 10 mm rat sciatic nerve gap was repaired with 14 mm long fibrin conduit enriched with different fillers for 12 weeks. - Spinal motoneurons retrograde labeling was used to assess the number of motoneurons regenerationg axons. - Morphological analysis (IHC) on nerve and on muscle.	- After 3 months axon regeneration and a reduction in muscle atrophy were observed. - The mean area and diameter of slow type muscle fibers were not statistically different between experimental groups.
	
	Wang et al., 2018b	Fibrin glue was pulled into a specially designed compactor with a silicone inlay around a stainless steel core.	- 5 mm rat nerve sciatic nerve gap repaired with fibrin conduit for 2 and 4 weeks to test fibrin adhesive characteristics. - Morphological analysis (macroscopical evaluation, IHC). - Morphometrical analysis to count axon number.	- Sutureless nerve repair with fibrin conduit fails to maintain nerve connections. - Fibrin conduit protected the regenerating axons from neuroma formation.

Among gelatin crosslinkers, genipin has been often used in tissue engineering applications. Genepin has low cytotoxicity and it is of natural origin, it can be obtained from geniposide isolated from the fruits of *Gardenia jasminoides* ELLIS (Cenni et al., 2000; Sung et al., 2001). Different gelatin-based conduits were produced using it as cross-linker. Chen Y. S. et al. (2005) used a genepin cross-linked gelatin conduit to repair a 10 mm rat sciatic nerve defect for 8 weeks confirming conduit biocompatibility, but with controversial results: nerve regeneration inside the conduit was not compared with a positive control group as autograft and they observed that after 8 weeks most of the regenerated axons were not myelinated. The authors also showed that the biomaterial starts to degrade 6 weeks from the repair and after 8 weeks the fragmentation is clear (Chen Y. S. et al., 2005). Four years later, the same authors showed that an increased conduit porosity can implement its performance, with good regeneration in the rat model, giving real perspective for future studies in longer defects (Chang et al., 2009).

Another natural cross-linking agent, proanthocyanidin, which is usually used as an antioxidant, was used to stabilize a gelatin conduit, reducing its degradation rate. The proanthocyanidin cross-linked gelatin conduit presents a rough outer surface and was used to repair a 10 mm nerve gap and the regeneration was assessed 8 weeks after the repair (Liu, 2008). The conduit was tested for its biocompatibility and degradation: gelatin and proanthocyanidin release was observed and it seems to support Schwann cell adhesion and growth. After 8 weeks *in vivo*, the conduit was well integrated into the surrounding tissues, holding its shape intact and no inflammatory reaction was observed. Histological and electrophysiological measurements were assessed and the regenerated units containing unmyelinated and myelinated axons were abundant.

Gámez et al. (2003) prepared a biodegradable gelatin-based nerve conduit using photoreactive gelatin able to be cross-linked upon visible-light irradiation. The conduit 15 mm long was implanted between the proximal and distal stump of a 10 mm rat sciatic nerve gap up to 12 months (Gámez et al., 2003). The photo-fabrication of the gelatin conduit allowed to obtain a conduit with controlled features such as length, wall thickness and inner diameter. The hollow conduit performances were compared with those of the same conduit enriched with fibers alone or impregnated with bioactive molecules (Gámez et al., 2004). Despite nerve regeneration was better in the two enriched experimental groups, morphological, functional, and electrophysiological response recovery were observed for all experimental groups.

Recently, the biocompatibility of a gelatin conduit cross-linked with bisvinylsulfonemethyl was assessed. Nerve regeneration at 8 weeks in a 10 mm rat sciatic nerve defect repaired through this conduit was similar to that obtained using a silicone guide previously tested. Gelatin cross-linking with bisvinylsulfonemethyl reduces gelatin swelling and improves its mechanical properties; moreover, unlike other gelatin conduits, this nerve guide is transparent (Ko et al., 2017).

A different study exploited gelatin proper characteristics to be used as protection around a rat sciatic nerve repaired with end-to-end technique. This scaffold possessed low mechanical strength: it collapsed and adhered to the repaired nerve, like a sheath. It was observed that the use of this scaffold around the nerve reduces scar formation, slows down inflammatory cell arrival, and shows better results in comparison to end-to-end repaired nerve unprotected by gelatin (Tao et al., 2017). To avoid gelatin tube collapse, recently a gelatin based-tube was stabilized with titanium micro-rods (Uranues et al., 2020). This conduit was tested for 8 weeks in mini-pig models to repair 6 mm sciatic nerve gaps and results similar to those obtained with a direct coaptation of the same gap were obtained at morphological, electrophysiological and functional level. Nevertheless, despite the conduit does not generate any immune response, titanium is synthetic and not biodegradable, thus reducing the attractiveness toward a gelatin conduit which is otherwise of natural origin and biodegradable.

Indeed, due to its weak mechanical strength and its rapid degradation, most of the gelatin-based scaffolds have been prepared using gelatin combined with other biomaterials of different origins, such as chitosan (Nie et al., 2014) or bioglasses used to produce conduits (Koudehi et al., 2014); or poly-L-lactic acid (PLLA) (Binan et al., 2014) and polycaprolactone (PCL) (Vatankhah et al., 2014) to obtain electrospun mats. Thanks to its chemical properties and its degradation kinetics, the gelatin scaffold can easily incorporate bioactive molecules which can be gradually released during scaffold degradation (Chen P. R. et al., 2005).

#### Silk Fibroin

Silk proteins are synthesized in the silk glands of arthropods like spiders and silkworms, and during metamorphosis it is spun in fibers. The main source of silk fibroin for human applications is silkworm, in particular *Bombyx mori*, which is extensively used in the textile industry and allows obtaining a high silk yield in comparison to spiders. Moreover, spider silk use is restricted for the less silk yield, for their cannibalistic behavior and their heterogeneity in nature (Pérez-Rigueiro et al., 2005; Omenetto and Kaplan, 2010; Radtke, 2016). Silk fibroin use is economically advantageous, in comparison to other natural-based biomaterials, because of its easy and cheap purification processes and its large-scale availability exploiting silk industry infrastructures (Kundu et al., 2013).

Silk fibroin possesses characteristics that suggest its use in biomedical applications: it contains repeated aminoacidic sequences, which determine the formation of a high number of β-sheets, which confer good mechanical properties (i.e., elasticity, flexibility, and high resistance to fracture and compression) and influence its degradation. Silk is biodegradable and it is slowly degraded by proteolytic enzymes (Altman et al., 2003; Yang et al., 2007a; Cao and Wang, 2009; Keten et al., 2010); its degradation is also influenced by silk processing methods: silk with a high crystal content ensures a low degradation rate (Wang et al., 2008c; Li et al., 2019). Nevertheless, these characteristics are attributed to native silk fibers, while silk derived from silk fibroin solutions are generally weak and fragile.

Silk fibroin biocompatibility is well established as silkworm-derived silk is widely used in clinical practice for surgical sutures (Cao and Wang, 2009). The absence of cytotoxicity was demonstrated both *in vitro* and *in vivo*. Studies on dorsal root ganglia, primary cultures of Schwann cells and embryonic rat hippocampal neurons showed no deleterious effects (Yang et al., 2007a; Tang et al., 2009; Zhao et al., 2013).

Nerve conduits made by silk fibroin obtained from both spiders and silkworms were able to bridge peripheral nerve gaps and to guide nerve regeneration. Although different studies confirmed that spider silk conduits promote nerve regeneration (Allmeling et al., 2008; Radtke et al., 2011; Huang et al., 2012), here we focused our attention on *Bombyx mori* derived silk fibroin, since it is the most available source. Silk conduits with different characteristics were tested over time with success (Table 3). Yang et al. (2007b) developed a silk fibroin conduit with oriented filaments inside which gives results close to those obtained with autograft in bridging a 10 mm rat sciatic nerve gap. This conduit is biocompatible, showing no signs of adverse immune response 6 months after the implantation, it is porous and its pore size is appropriate to allow nutrient exchange and to block unwanted cell inflow; it also possesses good mechanical strength, it is resistant to the surrounding muscular contraction and maintains its original shape. The conduit starts to be degraded 6 months after implantation.

Despite Yang’s conduit was obtained through freeze-drying processing, Park et al. (2015) produced a porous silk conduit through the electrospinning technique. This guide possesses mechanical characteristics similar to the freeze-drying conduit, but its wall is thinner and the conduit production method is easier. The electrospun conduit was used to bridge 10 mm nerve defects and a restore in motor function was observed 10 weeks after the repair in a rat model.

Multilayered silk conduits were produced and tested to obtain suitable characteristics for a nerve regeneration comparable with those obtained with gold standard repair techniques. Belanger et al. (2018) developed a tri-layered silk conduit, composed by two perpendicular layers of aligned fibers outside and one inner layer of random fibers, and compared its performance in nerve repair to a direct suture to repair 5 mm sciatic nerve defect. The device structure increases its inner surface area, improving axonal regrowth in terms of myelin thickness and axon diameter 8 weeks after the repair, even if no significant differences are observed in comparison to end-to-end repaired nerves. Nevertheless, conduit use is favorable since it reduces nerve tensioning during regeneration in comparison to end-to-end repair technique; moreover, this conduit is also easy to suture and possesses an optimized surface architecture and good mechanical properties.

Another tri-layered silk scaffold, used to repair a 10 mm gap and compared with autograft, consists of a textile layer interposed between two electrospun layers with controlled wall thickness and porosity (Alessandrino et al., 2019). *In vitro* assays carried out with Schwann cell and motor neuron cell lines demonstrated its biocompatibility, which was also confirmed by *in vivo* experiments: no inflammatory response and scar formation around the implant were observed 2 weeks after the repair. This short-term study showed early cellular colonization and a progressive axonal regrowth.

Since native silk confers good tensile and mechanical properties to conduits, and conduits produced with fibroin solutions do not possess the same characteristics, silk fibroin obtained from fibroin solutions could be blended with different biomaterials to reach adequate mechanical properties (Wang and Cai, 2010; Xu et al., 2016). Blending silk fibroin with synthetic biomaterial could also be a strategy to improve synthetic conduit performance and biocompatibility, since silk fibroin could stimulate fibroblast proliferation and VEGF secretion, which results in improved angiogenesis inside the conduit, proven to positively influence nerve regeneration (Wang et al., 2018a). Silk fibroin could also be blended with natural biomaterial such as collagen, indeed a silk fibroin-collagen nerve conduit, on which adipose-derived stem cells were co-cultured with Schwann cells, showed good results with nerve regeneration acceleration (Xu et al., 2016).

#### Fibrin

Fibrin is a fibrillar protein formed during blood clotting. Fibrin is mainly involved in hemostasis, but it plays a role in wound healing by forming a temporary matrix (Park and Woo, 2018).

Due to its role in hemostasis, it is widely used in clinical practice as a surgical glue (Albala and Lawson, 2006) and it has also been used to join nerve stumps with successful results (Ornelas et al., 2006a, b). Fibrin glue is biocompatible, and can be obtained from donors or can be acquired by different companies for clinical practice (Le Guéhennec et al., 2004), nevertheless, it can be obtained from the patient since it has been demonstrated that cell survival is better on autologous fibrin scaffolds (De la Puente and Ludeña, 2014).

The mechanical properties of this biomaterial are easily and highly tunable varying fibrin concentration and processing temperatures (Bruekers et al., 2016). However, since fibrin degradation rate is high, antifibrinolytic agents are necessary to prevent the conduit lysis (Rowe et al., 2007).

As well as for its high biocompatibility, fibrin can be used as a scaffold in tissue engineering thanks to its versatility, indeed its dissolving and coagulation characteristics can be modified by changing the dilution (Bensaïd et al., 2003; Fang et al., 2004). Thanks to fibrin adhesive characteristics, this type of conduit could be not sutured; nevertheless, a recent study demonstrated that sutureless nerve repair with fibrin conduit fails to maintain nerve connections due to poor mechanical stretch resistance (Wang et al., 2018b).

Different fibrin conduits were tested over time with success (Table 3). Kalbermatten et al. (2009) demonstrated the effectiveness in rat sciatic nerve regeneration of a conduit made by fibrin glue to repair 10 mm defects. Also, the fibrin conduit demonstrated a better axon regeneration length in comparison with PHB conduits 2 weeks after the repair and after 28 days axon full crossing was observed. This conduit does not collapse, supports axonal sprouting and is porous, allowing nutrient inflow, but at the same time its surface prevents fibrous tissue formation (Takazawa et al., 2005). Recently, the same fibrin conduit 14 mm long, filled with fibrin matrix or fibrin matrix containing human mesenchymal stem cells, was used to repair a 10 mm gap in rat sciatic nerve and after 3 months axon regeneration and a reduction in muscle atrophy were observed (McGrath et al., 2018), confirming its effectiveness in nerve repair promotion.

#### Keratin

The use of keratin as a biomaterial was the focus of several researches in the last four decades. “Hard” keratin proteins can be isolated from protective tissues such as hooves, nails and hairs, that contain structural proteins that are more resistant than “soft” keratin derived from cytoskeletal elements found in epithelial tissues (Hill et al., 2010). Human hair is a highly ornate superstructure composed of fibers that are self-assembled in the follicle, that are regulated by more than 30 cytokines and growth factors (Lyons et al., 1990; Jones et al., 1991; Hardy, 1992; Blessing et al., 1993; Stenn et al., 1994; Motonobu et al., 2013).

Keratin was demonstrated to be biocompatible, biodegradable, bioactive and it possesses a hydrophilic surface, which is absent in many synthetic polymers. Nevertheless, the practical use of keratin-based biomaterials is limited due to its poor physical and mechanical properties (fast degradation rate and low molecular weight) that can be improved using various cross-linking agents (Rajabi et al., 2020). Keratin itself has too weak mechanical resistance to be used alone as a nerve conduit, indeed it is largely used as conduit filler.

Because of its neuroinductive capability (Apel et al., 2008; Pace et al., 2013), human keratin has proven to be effective in promoting nerve regeneration in short gaps (5–15 mm) if used as hydrogel-filler for conduits in mouse (Apel et al., 2008; Sierpinski et al., 2008), rat (Lin et al., 2012; Pace et al., 2013), rabbit (Hu et al., 2003; Hill et al., 2011), and macaque models (Pace et al., 2014).

Besides, Gupta and Nayak used keratin as a protein source for scaffold fabrication and they succeeded to produce a keratin/alginate scaffold for tissue engineering applications. This scaffold was never used *in vivo* for peripheral nerve repair assessment, even if *in vitro* experiments reported promising results (Gupta and Nayak, 2016).

### Polyesters

Polyesters are natural biodegradable biopolymers, which can be obtained from renewable resources; among them the polyhydroxyalkanoates (PHA) family is the most successful one in tissue engineering applications (Anjum et al., 2016; Basnett et al., 2017). PHAs, which possess properties similar to the conventional petrochemical polymers, are usually produced by bacterial and archaeal fermentation in conditions of nutrient depletion or excess of carbon sources and are accumulated within their cytoplasm (Chen, 2010; Rodriguez-Contreras, 2019). PHA family members differ widely in their structure and properties depending on the type of microorganism, biosynthesis conditions, and on which carbon source was used during the production process (Licciardello et al., 2019; Mozejko-Ciesielska et al., 2019).

The specific advantage of PHA is its stable local pH during degradation, which improves its biocompatibility in comparison to other biomaterials used for medical devices (Koller, 2018). Nevertheless, PHA production has high costs, which could be reduced by different strategies, which include the development of recombinant microorganisms able to produce large PHA amounts, as recently reviewed by Zheng et al. (2020).

Poly (3-hydroxybutyric acid) and poly (3-hydroxybutyric acid-co-3-hydroxyvaleric acid) are the most widely studied members of this family. These polymers can be easily processed to obtain conduits using different production methods such as extrusion, electrospinning and rolling sheets on a mandrel (Arslantunali et al., 2014) and several preclinical studies on nerve regeneration using these PHA-based conduits were carried out with successful results (Table 4).

**TABLE 4:** Relevant studies on polyester based-conduits.

	References	Method of conduit production	Analyses	Results
**PHB**	Masaeli et al., 2012	Electrospinning and salt-leaching procedures.	- Conduit evaluation of mechanical and physical properties (tensile strength and modulus, dynamic contact angle and porosity). - *In vitro* analysis.	- The salt-leached scaffolds showed more wettability and permeability, but inferior mechanical properties. - Nanofibrous scaffolds can be a better substrate for cell attachment and morphology.
	
	Mohanna et al., 2005	Conduit obtained from PHB sheets (Astra Tech, Göteborg, Sweden), consisting of compressed PHB fibers	- Macroscopical observation. - Morphological evaluation of nerve regeneration 120 days after the repair of 2 or 4 cm rabbit peroneal nerve gaps. - Evaluation of atrophy and loss of muscle mass percentages.	- At harvest, conduits were covered by a fibrous pseudo-capsule and were still well vascularized. - No macroscopic evidence of tissue inflammation were observed. - The PHB tubes were flexible, firm in consistency and non-friable. - Regeneration had occurred across the 2 and 4 cm gaps in all animals.
	
	Young et al., 2002	Conduits obtained from PHB sheets (Astra Tech, Göteborg, Sweden), consisting of compressed PHB fibers	- Macroscopical observation. - Morphological evaluation of nerve regeneration up to 63 days after 4 cm rabbit peroneal nerve defect repaired with PHB conduit.	- At all harvest points the PHB tubes were found to be covered by a very thin pseudo-capsule. - From 21 days onwards, it was macroscopically evident that the PHB tube had become well vascularized. - The regenerating nerve fibers were aligned to the long axis of the PHB conduit. - An empty PHB conduit may not be sufficient to sustain optimal peripheral nerve regeneration of 4 cm gap.
	
	Hazari et al., 1999	Conduits obtained from PHB sheets (Astra Tech, Göteborg, Sweden), consisting of compressed PHB fibers	- Morphological evaluation of nerve regeneration 30 days after repair of 10 mm nerve gap in rat sciatic nerves.	- Good angiogenesis was observed at nerve ends and through the conduit wall. - Low level of inflammatory infiltration and a good nerve regeneration were observed in PHB conduit. - The rate and amount of nerve regeneration in PHB conduits did not fully overlap with that observed in a nerve graft.

**PHBV**	Biazar et al., 2013c	Nanofibrous electrospun sheets rolled around a cylindrical rod and sealed with heat to obtain a conduit.	- SEM on the conduit. - 30 mm rat sciatic nerve gap repaired with nanofibrous PHBV conduit for 4 months. - Macroscopical evaluation. - Morphological analysis (light microscopy and IHC) for nerve and muscle samples.	- Macroscopically a restore of nerve continuity was observed. - Similar skeletal muscle reinnervation and myelinated nerve fiber diameter distribution were observed between PHBV conduit group and the autograft experimental group.
	
	Biazar et al., 2013b	Nanofibrous electrospun sheets rolled and sealed with heat to obtain a conduit.	- SEM on the conduit. - 30 mm rat sciatic nerve gap repaired with nanofibrous PHBV conduit for 4 months. - Different behavioral tests to assess motor (toe out angle, toe spread analysis, walking track analysis, extensor postural thrust, swimming test, open-field analysis) and sensory recovery (withdrawal reflex latency) were performed.	- Motor and nociceptive functional recovery was similar in both experimental groups (PHBV conduit or autograft).
	
	Karimi et al., 2014	PHBV conduit designed onto micropatterned silicon wafers.	- Conduit characterization (SEM, mechanical and physical properties evaluation). - 30 mm rat sciatic nerve gap were repaired with PHVB conduit enriched or not with Schwann cells for 4 months. - Macroscopical evaluation and morphological analysis on nerve and muscle samples. - Behavioral analysis to evaluate motor and nociceptive function restore sensory and motor function.	- The presence of Schwann and glial cells in regenerated nerves was observed. - Nerve continuity was restored and myelinated fibers were observed for both conduit experimental groups. - Motor and sensory function recovery was observed for both conduit experimental groups.
	
	Hu F. et al., 2017	Aligned nanofibrous PHBV-based conduits.	- 10 mm rat sciatic nerve defect was repair with the conduit alone or enriched with adipose-derived mesenchymal stem cells (ASCs) or FGF2-*miR*-218-induced ASCs for 10 weeks. - Macroscopical evaluation and morphological analysis (light microscopy and IHC). - Functional recovery analysis (Catwalk analysis and SFI).	- Sciatic nerves were successfully reconnected in all experimental groups. - The number of myelinated axons was reduced in the experimental group with ASCs. - FGF2-*miR*-218 approach in combination with the conduits lead to better functional recovery and an improved nerve regeneration.
	
	Demirbilek et al., 2015	PHBV conduit made by oriented nanofibers obtained through electrospinning technique.	- 10 mm rat sciatic nerve defect was repaired with the PHBV conduit for 1, 2, and 4 months. - Macroscopical observation and morphological assessment. - Functional and electrophysiological analysis.	- Nerve regeneration inside the conduit was similar to that observed with autograft, even if autograft group presents a better and faster regeneration. - No differences were detected in terms of axon number and myelin sheath thickness. - Regenerating nerves of the PHBV conduit group were more vascularized then those of the autograft group, 8 weeks after the repair. - Functional recovery was observed for all experimental groups.

#### Poly (3-Hydroxybutyric Acid) (PHB)

Poly (3-hydroxybutyric acid) is the main polymer of the PHA family (Zhuikov et al., 2020). It is an innovative product of natural origin, obtained from bacterial fermentation, with potential characteristics including biodegradability and biocompatibility. Its promising applications as a biomaterial in different fields of tissue engineering (Wu et al., 2009; Bagdadi et al., 2018) and of peripheral nerve regeneration have been documented (Young et al., 2002). PHB conduits possess good mechanical and physical features, which can be modified by different production methods (Masaeli et al., 2012). Moreover, they are biodegradable, even if reabsorption time is long (over 2 years) and show early vascularization after implantation (Mohanna et al., 2005). PHB stability could be a disadvantage for short-term applications, but it is useful for long-term nerve reconstructions.

The PHB has been used as an alternative to direct epineural repair (Åberg et al., 2009) and to bridge short and long nerve defects. PHB conduits have been successfully used to repair a 10 mm gap in rat sciatic nerves up to 30 days; low levels of inflammatory infiltration were detected, macrophage levels were similar to those of rat nerves repaired with autograft and a good axon regrowth was observed. No failure in nerve regeneration was observed at all (Hazari et al., 1999).

For long nerve gaps, PHB conduits were tested on rabbits. Using the rabbit common peroneal nerve model, empty PHB conduits effectiveness was assessed across 2, 3, and 4 cm nerve defects up to 63 days. This study demonstrated good nerve regeneration across long gaps (Young et al., 2002). Nevertheless, regeneration through a hollow guide for long gaps can be enhanced through the addition of trophic factors, such as the glial growth factor/neuregulin1 (GGF/NRG1), which was resuspended in an alginate hydrogel used to bridge 2–4 cm gaps in rabbit common peroneal nerve with successful results both in short- and long-term (120 days) experimental conditions (Mohanna et al., 2003, 2005). These data suggest that PHB nerve conduits are suitable for long-gap nerve injury repair.

Due to their success in nerve regeneration, PHB conduits had been also used to compare fibrin glue conduits (Kalbermatten et al., 2009) and afterward the same authors also used a PHB conduit to evaluate the potentiality of an internal filler composed by a fibrin matrix loaded with Schwann cells or differentiated mesenchymal stem cells to repair 10 mm gaps in rat (Kalbermatten et al., 2008b). As an alternative to PHB nerve guides, PHB strips seeded with Schwann cells were used to bridge a 10 mm sciatic nerve gap in rats, since strips can provide a direct contact among the biomaterials, cells and surrounding tissues. PHB strips seem to support the early stages of nerve regeneration and show a fast regeneration 2 weeks after the repair (Kalbermatten et al., 2008a). More recently, the same authors tested PHB strips seeded with Schwann cells or with adipose-derived stem cells in a long-term study (12 months). Animals treated with enriched strips showed a better functional recovery in comparison with control animals treated without cells, with a higher number of axons reaching the distal stump and reduced muscle atrophy (Schaakxs et al., 2017).

Since chitosan reduced PHB crystallization, these biomaterials were successfully used in combination to produce aligned fibers by electrospinning technique to produce scaffolds for nerve tissue engineering (Karimi et al., 2018). Synthetic biomaterials such as polycaprolactone were also successfully used to be blended with PHB, to produce nerve guides with requested characteristics (Hinüber et al., 2014).

#### Poly (3-Hydroxybutyrate-Co-hydroxyvalerate) (PHBV)

Since PHB has some disadvantages, such as fragility, lack of hydrophilicity and tendency to crystallize, characteristics which reduce flexibility and ductility of the conduit, the copolymer PHBV was made to compensate these disadvantages of PHB in order to improve the physic-chemical properties of that biomaterial (Zhuikov et al., 2020). In this way, mechanical properties can be modulated by co-polymerization of PHB with hydroxyvalerate to form PHBV biopolymer (Koller, 2018), considered more flexible and easier to process, even if it presents some disadvantages such as a narrow processing window and a low strain-at-break in comparison to petroleum-based synthetic polymers (Zhao and Turng, 2015).

Biazar et al. (2013c) used a PHBV fibrous tubular conduit produced by electrospinning technique to repair a 30 mm gap in rat sciatic nerve with satisfactory results within 4 months, with skeletal muscle reinnervation and a myelinated nerve fiber diameter distribution similar to that of the autograft experimental group. Beyond its high porosity (about 95%) and biocompatibility, this conduit is easy to handle and to suture; it is flexible and can also be bent up to 180° and then it restores its original shape. The histomorphological study and behavioral tests confirmed and strongly supported the use of this conduit to repair 30 mm nerve gaps (Biazar et al., 2013b). The same experiments performed with PHBV fibrous conduit were replicated with the introduction of Schwann cells inside the conduit, confirming its effectiveness (Biazar and Heidari Keshel, 2013; Biazar et al., 2013a).

PHBV efficacy in nerve repair was also tested through the production of a PHBV conduit made by micropatterned wafers (Karimi et al., 2014). The authors observed, in rat sciatic nerve, sensory and motor function recovery 4 months after a 30 mm gap repair with a PHVB conduit enriched or not with Schwann cells.

Also aligned nanofibrous PHBV-based conduits were tested to repair nerve injuries. Hu F. et al. (2017) repaired 10 mm rat sciatic nerve defect up to 10 weeks, with aligned electrospun nanofibres conduit alone or enriched with adipose-derived mesenchymal stem cells (ASCs) or FGF2-*miR*-218-induced ASCs, demonstrating that FGF2-*miR*-218 induction approach combined with the presence of the PHBV scaffold improves nerve regeneration. In another study, the PHBV biopolymer was derived from *Alcaligenes eutrophus* using a nitrogen-rich medium and an excess of carbon source. A conduit composed of PHBV oriented nanofibers, used to repair a 10 mm nerve gap, was obtained by electrospinning technique and, to prevent a collapse, the conduit was filled with a drop of 1% agarose (Demirbilek et al., 2015). Nevertheless, the PHBV tendency to collapse was not observed in the other studies reported in this paragraph, even in presence of longer gaps. Functional and morphological analyses were performed 2 and 4 months after the repair (functional recovery was also tested after 1 month) and it was demonstrated that nerve regeneration is similar to that observed with autograft, even if the autograft group presents a better and faster regeneration in comparison to the PHBV conduit group (Demirbilek et al., 2015). Interestingly, regenerating nerves of the PHBV conduit group were more vascularized then those of the autograft group 8 weeks after the repair; the presence of blood vessels should sustain nerve regeneration by allowing nutrient inflow.

A nanofibrous PHBV conduit cross-linked with chitosan was obtained and showed its suitable physical, mechanical, and structural properties to promote nerve regeneration after a 10 mm gap repair, up to 4 months. The conduit was also enriched with Schwann cells, which adhere well to the fibrous scaffold (Biazar and Keshel, 2013).

## Future Trends in the Design of NGCs Using Natural-Based Biomaterials

Conventional processing technologies associated with surface modification approaches have been successfully applied in the NGC development so far (Chiono and Tonda-Turo, 2015). Natural-based biopolymers were processed mainly through the casting of porous membranes and subsequent wrapping, electrospinning and dip-molding technologies to obtain channels. However, these methods do not allow to obtain some suitable mechanical characteristics or to obtain complex geometry as well as to encapsulate biological cues such as growth factors and nerve supporting cells into the conduit to enhance the regeneration process. Recently, non-conventional technologies have raised interest as an alternative to process natural-based polymers to form NGCs (Johnson and Jia, 2016). Among others, 3D bioprinting offers unique features to allow customized geometry via clinical imaging of damaged nerve including biological cues within the fabrication process enhancing the effectiveness of the biological stimuli after *in vivo* implantation. Maiti and Díaz (2018) have recently revised the use of bio-inks as forming materials for the development of NGCs, highlighting the superiority of natural-based biopolymers compared to synthetic ones. Furthermore, recent results reported the possibility to produce nerve guidance channels as well as lumen loading cells and the establishment of growth factor gradients along the length of a nerve guide using 3D bioprinting (Petcu et al., 2018). On the other hand, recent innovations on biomaterials design are being directed toward the use of modified natural polymers to confer improved mechanical properties (Noè et al., 2020; Tonda-Turo et al., 2020). The use of methacrylate natural polymers is currently wildly applied to form complex geometries using additive manufacturing. Among others, methacrylated gelatin (GelMA), thanks to its biocompatibility and mechanical tunability, has been applied in many fields of tissue engineering and NGC having multiple channel geometries have been produced through 3D bioprinting of GelMA (Ye et al., 2020). 3D printed GelMA hydrogels have been tested as nerve conduits showing promising results. The high hydrophilicity of this biomaterial allows the incorporation of nanoparticles (Tao et al., 2019; Xu et al., 2019) or platelets (Tao et al., 2020b) to increase peripheral nerve regeneration and functional recovery.

## Conclusion

Conduits obtained from natural-based biomaterials share many characteristics with synthetic ones, which are also suitable for their use in peripheral nerve regeneration. Nevertheless, the advantage of natural biopolymers is not only their higher biocompatibility, but more relevant is the fact that these polymers can be extracted by different renewable sources and some of them can be obtained from wasted food. Currently, renewable source use is of fundamental interest for the world populations to enhance sustainability, environmental protection and to preserve human well-being. Even the patient itself could be the source of the biomaterial for the conduit production, like in the case of fibrin and keratin, thus reducing the risk of rejection and increasing cell survival and nerve regeneration.

Another important aspect to consider is that natural-based biomaterials are bioactive. In the literature the attention is mainly focused on the bioactivity effect on nerve regeneration, suggesting that the release of neurotrophic factors or the presence of bioactive binding sites inside a conduit positively influences nerve regeneration. Synthetic materials hold the promise that their properties can easily be tuned and controlled; however, they lack sites for specific proteins to bind and cells to interact with, so there is insufficient integration with the native tissue. Thus, different strategies are used by researchers to improve conduit performance, such as the introduction of internal filler hydrogels or cells releasing neurotrophic factors or the direct modification of the conduit. Some conduits are directly modified to obtain a covalent attachment of biochemical cues such as proteins, peptide sequences, and growth factors to improve their bioactivity. Natural materials have the advantage of conferring the needed biological sites for proteins to bind and biological cues for cell behavior to be controlled. This bioactivity is less under control in comparison to that of synthetic biomaterials, nevertheless, in most cases, as previously reported, the release of molecules, such as chito-oligosaccharides (Gong et al., 2009; Zhao et al., 2017), improves nerve regeneration without interfering with other biological processes. In the literature, most of preclinical studies pay more attention to nerve regeneration inside the conduit and biomaterial local effects in the implantation site rather than on possible systemic consequences. Indeed, few studies consider the systemic effect of the biomaterials, which could be a strategy to address the problems of the non-controlled multi-bioactivity of the different biopolymers. Nevertheless, researchers tend to consider as a good characteristic the presence of bioactive sites on natural biopolymers, rather than consider it as a problem. Furthermore, some authors believe that a natural biomaterial, such as silk, requires to be modified through different strategies, to increase its bioactivity (Magaz et al., 2018).

As well as biomaterials in general, natural-based biomaterials described in this manuscript present advantages and disadvantages (Table 1), and some of them are more suitable for being used as nerve conduits or as internal filler of the conduits. To overcome natural biomaterial limitations, a successful strategy is biomaterial blending. The combination of natural-based biomaterials with synthetic ones can enhance the poor mechanical characteristics of the natural ones, while thanks to their higher biocompatibility, natural biomaterial blending reduces inflammatory response induced by synthetic materials (Nectow et al., 2012). Also, natural-based biomaterial blending could be a successful strategy since it could allow using natural biomaterial commonly used as an internal filler (as alginate) to obtain conduits, exploiting the mechanical strength of other ones like chitosan (Pfister et al., 2007a).

Finally, it appears increasingly clear that a universal ideal conduit is difficult to be produced since conduit properties have to be evaluated case by case according to the gap length, but also on the implant anatomical district. Also, for conduit wall fitting nerve diameter should be taken into account and nerve elasticity changes depending on nerve size (∼21,188 Pa for a pig sciatic nerve and ∼10,910,000 Pa for a human median nerve) and on how distal the nerve lesion is from the spinal cord (Stouthandel et al., 2020). Then, it must be considered that when Young’s modulus is reported in the validation studies of a biomaterial, only partial information is given referring to the NGC outer layer mechanical behavior, a characteristic which can be modified by the biomaterial dryness (De Masi et al., 2019) and by the concentration and type of fillers that generally increase the NGC stiffness (Ryan et al., 2017; Tonda-Turo et al., 2017a; Vijayavenkataraman et al., 2019).

In addition, in preclinical studies other information about conduit manufacturing and some details about conduit characteristics are often missing, such as wall thickness or porosity, which could be useful to understand how nerve regeneration is influenced by conduit properties. Furthermore, the conduit properties often are not described with quantitative parameters that could allow standardization of criteria and protocols.

Moreover, even if some characteristics of an ideal conduit are well outlined, such as biocompatibility, other more complex like conduit degradation or wall thickness, which influences conduit permeability and porosity, are non-solved required properties, which need further investigations. Consequently, despite conduits showed to efficiently support nerve regeneration and are often successfully used, nerve autografts continue to be the gold standard technique for repairing long nerve defects, thus highlighting the need to develop more effective alternatives.

## Author Contributions

BF, GG, and SR organized the manuscript. BF prepared the figures. BF and GC wrote different sections of the manuscript. All authors contributed to manuscript revision, and read and approved the submitted version.

## Conflict of Interest

The authors declare that the research was conducted in the absence of any commercial or financial relationships that could be construed as a potential conflict of interest.
